# Abnormality in GABAergic postsynaptic transmission associated with anxiety in *Bronx waltzer* mice with an *Srrm4* mutation

**DOI:** 10.1016/j.ibneur.2023.12.005

**Published:** 2023-12-25

**Authors:** Yuka Shirakawa, Heng Li, Yuki Inoue, Hitomi Izumi, Yoshimi Kaga, Yu-ichi Goto, Ken Inoue, Masumi Inagaki

**Affiliations:** a Department of Developmental Disorders, National Institute of Mental Health, National Center of Neurology and Psychiatry, 4–1-1 Ogawa Higashi, Kodaira, Tokyo 187–8553, Japan; b Department of Mental Retardation and Birth Defect Research, National Institute of Neuroscience, National Center of Neurology and Psychiatry, 4–1-1 Ogawa Higashi, Kodaira, Tokyo 187–8553, Japan

**Keywords:** Anxiety, GABAergic interneuron, Parvalbumin, Srrm4, *Bronx waltzer* mouse

## Abstract

The homozygous *Bronx waltzer* (*bv*) mouse, which shows hearing impairment, also exhibits anxiety accompanied by a reduction in cortical parvalbumin (PV)-positive GABAergic interneurons. Recently, a mutation in splicing factor Ser/Arg repetitive matrix 4 (Srrm4) was found in *bv* mice. However, the cellular consequences of the *Srrm4* mutation for anxiety remain unknown. Here, we tested our hypothesis that *bv* mutant primarily affects interneurons through a cell-intrinsic pathology that leads to a reduction of interneurons and consequently causes anxiety. We found that the anxiety becomes apparent at 6 weeks of age in *bv/bv* mice. However, in situ hybridization revealed that *Srrm4* is not expressed in interneurons, but rather dominates in pyramidal neurons. In addition, the PV-positive GABAergic interneurons were not reduced in number in the *bv/bv* cortex when anxiety became evident. However, electrophysiological abnormality of GABAergic transmission from interneurons was concomitantly present. Pharmacological blockage of GABA_A_ receptors revealed increased excitability in *bv/bv* mice, although no gross change occurred in the expression of an *Srrm4*-downstream gene, *Kcc2*, which regulates chloride flux upon GABAergic transmission. These findings suggest that the *bv*-associated *Srrm4* mutation mainly involves post-synaptic GABAergic transmission in the central nervous system, which may be associated with the anxiety phenotype in *bv/bv* mice.

## Highlights

• An *Srrm4* mutation causes anxiety-like behavior in *bv* mice.
• Reduced GABAergic interneurons seen in adult stage is absent when anxiety emerges.
• GABAergic interneurons are not major source of *Srrm4* expression.
• Altered postsynaptic GABA receptor transmission may underlie anxiety in *bv* mice.

## 1 Introduction

Anxiety is a major clinical phenotype commonly observed in many human neurodevelopmental and psychiatric disorders and of course anxiety disorders (Simonoff et al., 2008, Lever and Geurts, 2016, Braga et al., 2013, McNally, 2002, Ginzburg et al., 2010). Given that anxiety is linked to such a wide variety of clinical conditions, the neuronal circuits associated with this phenotype are likely involved in a broad range of brain abnormalities. Anxiety is regulated by a well-established circuit involving the amygdala, prefrontal cortex, and hippocampus (Tovote et al., 2015), and inhibitory GABAergic neurotransmission from interneurons plays a crucial role in this regulation (Tasan et al., 2011, Babaev et al., 2018).

A number of genetic and pharmacological models of anxiety have been reported, mainly targeting molecules and pathways associated with GABAergic neurotransmission in rodents. Mice deficient in the 65-kDa isoform of glutamic acid decarboxylase (GAD65) exhibit increased anxiety-like behaviors (Kash et al., 1999), and knockout mice lacking the urokinase-type plasminogen activator receptor, which has been associated with autism, display increased anxiety and exhibit region-specific reductions in PV-containing GABAergic interneurons in the cingulate and front-parietal regions of the cerebral cortex (Powell et al., 2003, Levitt, 2005, Eagleson et al., 2010). Moreover, positive modulators of GABAergic neurotransmission, such as benzodiazepines, have been shown to produce anxiolytic effects in rodent ethological models of anxiety (Rupprecht et al., 2009, Treit et al., 2010).

In our previous study, we found that homozygous *Bronx waltzer* (*bv/bv*) mice exhibit heightened levels of anxiety (Matsuda et al., 2011). Prior to that, this mouse was recognized as a model for hearing impairment, showing selective inner hair and pillar cell degeneration in the cochlea (Whitlon et al., 1996, Tucker et al., 1999). Consistent with this behavioral phenotype, *bv/bv* mice present with significant reductions in PV-containing GABAergic interneurons in some cortical areas at 3–4 months of age, as well as significantly reduced activity in cortical high-frequency electroencephalogram components (Matsuda et al., 2011). Therefore, we suggested that *bv* mice could be used as a model to clarify the GABAergic system-mediated neural basis of anxiety.

Recently, the mutation responsible for the *bv* phenotype was found in the splicing regulator Ser/Arg repetitive matrix 4 gene (*Srrm4*, also known as *nSR100*) (Nakano et al., 2012). Specifically, *bv* allele has a 2,710-bp deletion in the *Srrm4* gene, which removes a portion of the last intron and the entire coding region of the last exon, but leaves the polyadenylation site intact. The Srrm4 protein is a neuron-specific splicing regulator involved in various aspects of neuronal development and function. In vitro studies have shown that *Srrm4* knockdown in Neuro 2a cells impairs neurite outgrowth (Calarco et al., 2009, Ohnishi et al., 2017), while in vivo knockdown of *Srrm4* in mouse embryos prevented differentiation of neuronal progenitors in the cortex (Raj et al., 2011). Srrm4-deficient mice show defects in cortical layering and axon guidance (Quesnel-Vallières et al., 2015). In cultured cortical neurons lacking Srrm4, the density of excitatory synapses was increased, while that of inhibitory synapses was decreased (Quesnel-Vallières et al., 2016). Thus, Srrm4 may play some functional roles in the regulation of neuronal networks involving GABAergic interneurons. However, it is unclear how the *bv*-causing Srrm4 mutation affects GABAergic interneurons to cause anxiety.

Based on these findings, we hypothesized that *bv*/*bv* mutants primarily affect interneurons through a cell-intrinsic mechanism that leads to a reduction in interneurons and eventually causes anxiety. To validate this hypothesis, we sought to clarify the role of Srrm4 in postnatal GABAergic interneurons. Firstly, we examined developmental changes related to anxiety state in the *bv/bv* mouse. We then ascertained the postnatal expression pattern of *Srrm4* mRNA in the wild-type mouse brain. Next, we investigated when the reduction of GABAergic interneurons occurs in the postnatal *bv/bv* cortex. Finally, we assessed the functionality of GABAergic neurotransmitters in *bv/bv* mice using patch-clamp recording, the seizure susceptibility test, and evaluation of mRNA expression.

## 2 Experimental procedure

### 2.1 Animals

*Bv/bv* mice have been bred at the National Institute of Mental Health, National Center of Neurology and Psychiatry (NCNP) in Japan since 1998. Breeding *bv/bv* pairs were obtained through the courtesy of Dr. H. M. Sobkowicz (Department of Neurology, University of Wisconsin, Madison, WI, USA). The *Bv/bv* mice were back-crossed onto C57BL/6 J for more than 12 generations. Mice were housed individually on a 12/12-hour light/dark cycle, with ad libitum food and water, as well as constant temperature and humidity. All experimental protocols were conducted in accordance with the guidelines for animal care and regulated by the animal committee of the National Institute of Neuroscience.

### 2.2 Behavior experiments

The *bv/bv* mice on a C57BL/6 J background, as well as *bv/+* heterozygotes and wild type control mice, were used in the open-field exploration test and the elevated plus maze exploration test at 3 weeks (*bv*/*bv*; 2 males, 1 female, *bv*/+; 9 males (open field) or 8 males (elevated plus maze), wild-type; 8 males), 6 weeks (*bv*/*bv*; 6 males, *bv*/+; 4 males, wild-type; 5 males), and 3 months of age (*bv*/*bv*; 7 males, *bv*/+; 5 males, wild-type; 5 males). Most *bv/bv* mice in C57BL/6 J background do not show circling behavior, which usually becomes apparent around 4 weeks of age (Matsuda et al., 2011). Each experiment was recorded on videotape, and the data were transferred onto MPEG-format video files. For objective, quantitative evaluations of behavior, automated tracking was performed using EthoVision 3.1 software (Noldus Information Technology Inc., Leesburg, VA; Noldus et al., 2001). The coordinates of the animal were plotted in each frame.

#### 2.2.1 Open-field exploration test

Each mouse was place in the center of a 50 × 50 cm activity arena and allowed to explore for 5 min. As an activity parameter, the total distance moved was calculated.

#### 2.2.2 Elevated plus maze exploration test

The arms of the elevated maze were 50 cm long and 10 cm wide, with two of them enclosed in 40-cm-high opaque plastic walls. The maze was raised 50 cm from the floor. Each mouse was placed in the center of the maze and their behavior was recorded for 5 min

### 2.3 In situ hybridization

Sense or antisense *Srrm4* RNA probes, obtained from an 855-bp fragment of *Srrm4* cDNA referred from Allen Brain Atlas (Allen Institute for Brain Science, USA), was labeled with digoxigenin (Dig)-UTP were generated using the Dig RNA Labeling Mix (Roche).

Brains from C57BL/6 J mice at P1, P7, and P21 were fixed in 4% paraformaldehyde (PFA) in phosphate-buffered saline (PBS) at 4 °C, before coronal sections were prepared using cryostat at 25-µm thickness and collected onto MAS-coated glass slides. Pretreatment, hybridization, and probe detection were performed following the methods previously described (Ishii et al. 1998).

### 2.4 Immunohistochemistry

The *bv/bv* on C57BL/6 J background and control mice were used in the immunohistochemical analysis at 3 weeks and 8 weeks of age (n = 5 in each group). Under deep anesthesia, the mice were perfused transcardially with 0.1 M PBS, followed by 4% PFA in PBS. The brains were removed surgically, post-fixed in 4% PFA in PBS overnight at 4 °C, cryoprotected in 30% sucrose in PBS. Coronal sections were cut on a cryostat at 40-µm thickness and collected onto a MAS-coated glass slide (Matsunami Glass Industries Ltd., Osaka, Japan).

Immunohistochemical staining was processed as reported previously (Matsuda et al., 2011). The immunostained sections were observed and photographed with BZ-X710 (Keyence Co., Osaka, Japan). Visually-detected GAD67-expressing and GAD67/PV-co-expressing cells were counted in the ACC at bregma levels of + 0.5 mm, in the SC at bregma levels − 2.0 mm, and in the VC and AC at bregma levels − 2.7 mm. The cell count and area of the attractive region were analyzed using ImageJ software.

### 2.5 Slice preparation and patch-clamp recording

The *bv/bv* and wild type control mice were used in the whole-cell patch clamp procedure at 6–8 weeks of age (n = 5 in each group). Brain slice preparation and patch-clamp recordings were performed as reported previously (Sekiguchi et al., 2008). Briefly, in the recording chamber, the coronal brain slices (300 µm thick)were perfused using artificial cerebrospinal fluid (ACSF; 124 mM NaCl, 3 mM KCl, 2 mM CaCl2, 1.2 mM KH_2_PO_4_, 1.3 mM MgSO_4_, 26 mM NaHCO_3_, 10 mM glucose, pH 7.4, 295 mOsm.). Patch electrodes (resistance: 3–6 MΩ) were filled with the following solution: 105 mM potassium gluconate, 30 mM KCl, 10 mM Hepes, 0.5 mM EGTA, 1 mM MgCl_2_, 12 mM sodium phosphocreatine, 3 mM Mg-ATP, 0.5 mM Na-GTP, pH 7.3, at 295 mOsm. Whole-cell patch clamp recordings were obtained from the soma of pyramid-shaped neuron in voltage-clamp mode. The recordings were obtained from the ACC, SC, and BLA of both hemispheres. Three slices were used per mouse, and recordings were only performed on one neuron per slice. The electrophysiological signal was amplified and filtered at 5 kHz using a MultiClamp 700B patch-clamp amplifier (Axon Instruments, Union City, CA, USA). Data were digitized at 50 kHz and acquired using Clampex software (ver. 9.2; Axon Instruments). The access resistance was between 7 and 30 MΩ, and was monitored frequently to check for resealing.

Spontaneous and miniature GABAergic currents were studied under voltage clamping (−70 mV holding potential), with blockade of glutamatergic currents using 20 µM CNQX disodium and 10 µM MK-801. Miniature GABAergic currents were recorded in the presence of 0.5 μM TTX. The traces were analyzed using software (Mini Analysis Program; v.6.0.7, Synaptosoft, Decatur, GA, USA). The threshold amplitude for current detection was 5 pA, and the times required for a 37% decay and a 10–90% rise in the response were taken as the decay and rise times, respectively.

### 2.6 Seizure susceptibility test

The *bv/bv* and wildtype control mice were used in the seizure susceptibility test at 6 months of age (n = 5 in each group). After the mice were injected intraperitoneally with PTZ (20 mg/kg in saline), they were placed in a chamber and their seizure behavior was recorded by video.

### 2.7 RNA extraction and real-time PCR

Total RNA was extracted from the cortex using RNeasy Lipid Tissue Mini Kit (Qiagen, Hilden, Germany). First-strand cDNA was synthesized from total RNA using SuperScript III (Invitrogen, CA, USA). The cDNA samples were amplified using LightCycler 480 SYBR Green I Master (Roche, Basel, Switzerland) and detected using the Roche LightCycler 480 real-time PCR system. The mRNA levels were normalized relative to expression of GAPDH. The sequences of primers are described in Table 1.

**Table 1:** The sequences of primers used in real time PCR analysis.

Gene	Primer sequence (5′→3′)
*Nkcc1*	AGGAGCATTCAAGCACAGCTAACA
	CGCTCTGATGATTCCCACGA
*Kcc2*	GCGGGATGCCCAGAAGTCTA
	GATGCAGGCTCCAAACAGAACA
*Dlg4*	TCCGGGAGGTGACCCATTC
	TTTCCGGCGCATGACGTAG
*Gphn*	TGGTCCAGGGGATCGTTTCAT
	TTGTAACCCGCATCACTTGTC
*GAPDH*	CTGGAGAAACCTGCCAAGTA
	TGTTGCTGTAGCCGTATTCA

### 2.8 Statistical analysis

To analyze the behavioral data of the open-field exploration test and elevated plus maze exploration test, the Kruskal–Wallis test was used. If this test indicated significance, the Steel–Dwass test was used to detect significant differences among groups. The cell density data of the immunohistochemistry, the amplitude of the IPSCs, the time data of the seizure susceptibility test, and the mRNA expression data were analyzed using the Mann–Whitney U test. The Kolmogorov–Smirnov test was used to compare the cumulative probability distribution comparisons of mIPSCs and sIPSCs between the two data groups. To analyze correlation, the Spearman’s rank correlation coefficient (ρ) was calculated. A p-value < 0.05 was considered statistically significant. All statistical analyses were performed with R software version 3.5.1.

## 3 Results

### 3.1 *Bv/bv* mice present with anxiety-like behavior after 6 weeks of age

In our previous study, *bv/bv* mice exhibited heightened levels of anxiety at 3–4 months of age (Matsuda et al., 2011). However, it was unclear whether these anxiety-like behaviors were early developmental problems or rather late-onset phenomena. Therefore, in the present study, we examined the behavioral phenotype of *bv/bv* mice using the elevated plus maze paradigm test at the following time points: 3 weeks, 6 weeks, and > 3 months of age.

**Fig. 1:**
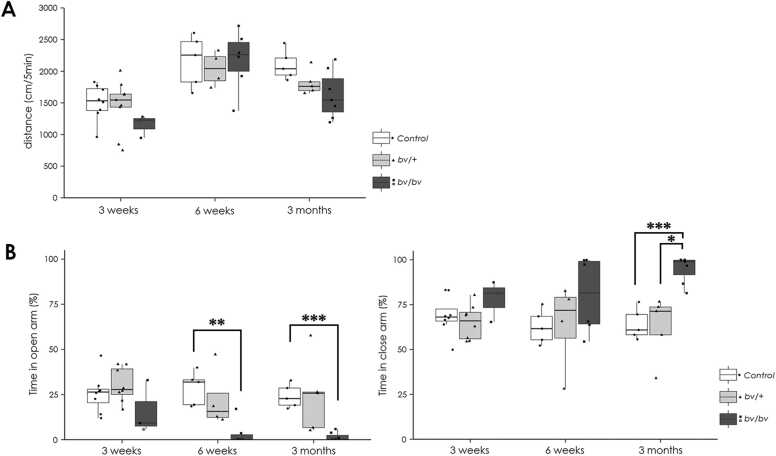
Locomotion data from the open-field exploration and elevated plus maze paradigm tests. (A) Developmental changes in total distance moved in the open-field exploration test in *bv/bv*, *bv/+* , and control mice at 3 weeks, 6 weeks and > 3 months of age. (B) Developmental change in the percentage of time spent in the open arms (left panel) and closed arms (right panel) of the elevated plus maze paradigm test for *bv/bv*, *bv/+* , and control mice at 3 weeks, 6 weeks and > 3 months of age. Within each box, the horizontal line indicates the median value, and the boxes extend from the 25th to 75th percentile of the value distribution for each group. Vertical lines indicate the largest and smallest data points within 1.5 between the 25th and 75th percentile quartiles for each group. The dot plots indicate data for each mouse. The only female of the 3 weeks old *bv/bv* mouse data is indicated by open square dot. * *p* < 0.05, * * *p* < 0.01, * ** *p* < 0.001.

Using the open-field platform, we verified that there was no difference in locomotion among *bv/bv*, *bv/+* , and wild-type mice (Fig. 1A). In the elevated plus maze, genotype showed an effect on distance in the open arms, which is an anxiety parameter, at 6 weeks (*p* < 0.01) and 3 months (*p* < 0.01) of age. Subsequent *post-hoc* analyses revealed that *bv/bv* mice advanced a significantly shorter distance in the open arms than control mice (at 6 weeks and 3 months of age) and *bv/+* mice (at 3 months of age; *p* < 0.05 in all cases; Fig. 1B). These results suggest that *bv/bv* mice already exhibit anxiety-like behaviors at 6 weeks of age and after, but not apparent at 3 weeks of age.

### 3.2 GABAergic interneurons are not a major source of the *Srrm4* expression

Srrm4 is expressed in both the brain and neural tube during early neurogenesis (Quesnel-Vallières et al., 2015) and peaking from E11 to E18 (Raj et al., 2011, Raj et al., 2014) in mice. Afterward, Srrm4 expression decreases as neuronal maturation occurs (Raj et al., 2014). However, the postnatal expression profile of *Srrm4* has not been fully defined (Lein et al., 2007). Therefore, we sought to determine the postnatal expression pattern of *Srrm4* mRNA using in situ hybridization on P1, P7, and P21.

**Fig. 2:**
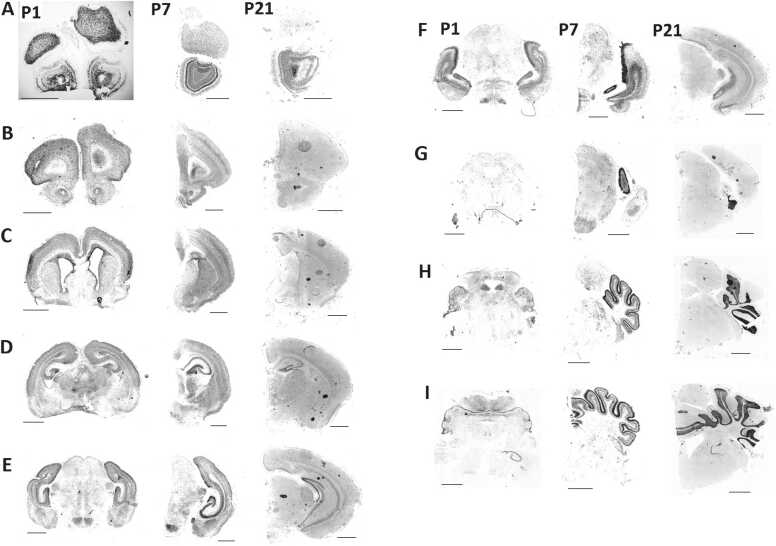
Developmental changes in expressions and distributions of *Srrm4* mRNA in the C57BL/6 J mouse brain. Adjacent coronal sections at postnatal day (P)1, P7, and P21 were examined using in situ hybridization. Scale bar indicates 1000 µm.

The postnatal expression of *Srrm4* mRNA in the mouse brain was distributed widely and exhibited no major developmental changes from P7 to P21 (Fig. 2). Throughout postnatal development, *Srrm4* mRNA was observed in the olfactory bulb, telencephalon, diencephalon, mesencephalon, and cerebellum. Among those areas, strong *Srrm4* expression was particularly observed in the olfactory bulb, hippocampus, and cerebellar cortex. Strong expression in the ventricular zone was observed on P1. In contrast, little or no expression was observed in the white matter and pons. Overall, the signal of Srrm4 was strong where neuronal density was high. Interestingly, in the diencephalon and mesencephalon, we observed relatively strong Srrm4 expression in the dorsal and ventral thalamus, dorsal lateral and medial geniculate nucleus, and mammillary nucleus; all of these sites reportedly show weak GAD67 expression (Tamamaki et al., 2003). Conversely, in the superior colliculus and substantia nigra pars reticulate, where GAD67 expression is relatively strong, the *Srrm4* signal was weak. These results suggest that regions enriched with Srrm4-expressing cells do not overlap with those enriched with GAD67-expressing GABAergic interneurons.

**Fig. 3:**
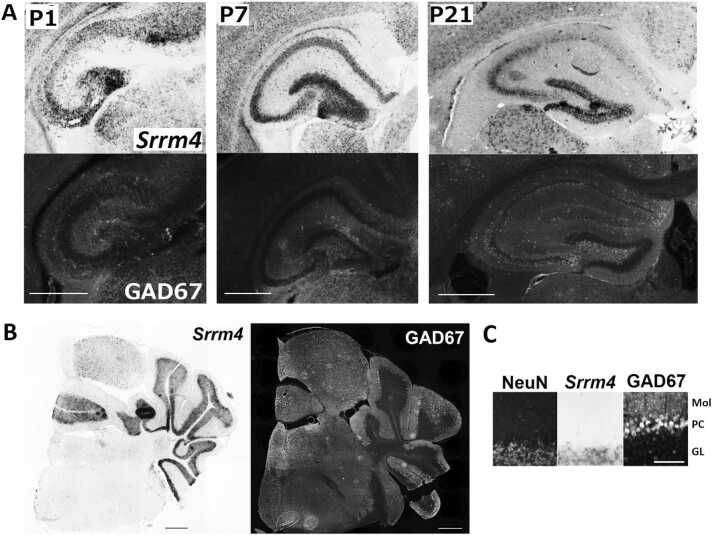
*Srrm4* mRNA, GAD67, and NeuN distribution in the hippocampus and cerebellum. (A) Hippocampus at postnatal day (P)1, P7, and P21. (B) Section of the cerebellum level at P21. (C) Close-up views in cerebellum. Scale bars in (A) and (B) indicate 500 µm. Scale bar in (C) indicates 100 µm. Mol, molecular layer; PC, Purkinje cell layer; GL, granule cell layer.

Furthermore, we investigated whether *Srrm4* expression overlapped with the expression pattern of GABAergic interneurons in the hippocampus and cerebellum (Fig. 3). In the hippocampus, *Srrm4*-expressing neurons were observed in the pyramidal and granule cell layers, where no GAD67-expressing cells were observed (Fig. 3A). In the granule cell layer of the cerebellum, where the Srrm4 signal was observed, no GAD67-expression cells were evident. On the other hand, in the molecular layer and Purkinje cell layer, where GAD67-expressing cells were present, *Srrm4* mRNA expression was absent (Fig. 3B&C). Lastly, the distribution of NeuN-positive granule cells overlapped with expression patterns of *Srrm4*. Together, these findings suggest that Srrm4 is mainly expressed in the excitatory neurons, and that GABAergic interneurons are not a major source of Srrm4 expression.

### 3.3 No reduction in GAD67 and PV-immunoreactive cells in postnatal *bv*/*bv* cortices

Using GAD67 and PV immunohistochemical analysis, we previously showed that PV-positive GABAergic interneurons are significantly reduced in the cerebral cortex of adult *bv/bv* mice (Matsuda et al., 2011). However, it remains to be determined whether this GABAergic abnormality is already present at birth or whether it occurs later in development. Thus, we quantitatively determined the number of GABAergic interneurons during postnatal development.

**Fig. 4:**
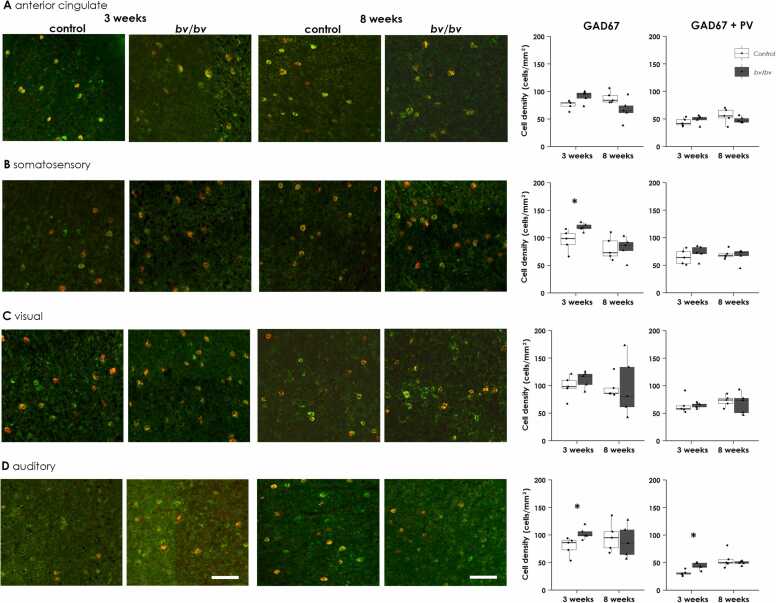
Immunostaining of neurons expressing GAD67 and PV. Anterior cingulate (A), somatosensory cortex (B), visual cortex (C), and auditory cortex (D) of *bv/bv* and control mice at 3 weeks and 8 weeks of age. Left four columns show merged microscopic images for GAD67 (green) and PV (red) co-immunostaining for each group and age. The cells co-expressing GAD and PV show orange in color. The boxplots (right two columns) show the density of GAD67-expressing and GAD67/PV co-expressing cells. The scale bars indicate 100 µm. * *p* < 0.05.

We examined the number of GAD67-positive and GAD67/PV-double positive interneurons in the anterior cingulate (ACC), somatosensory (SC), visual (VC), and auditory cortices (AC) of *bv/bv* and control mice at 3 weeks and 8 weeks of age (Fig. 4). At 3 weeks of age, *bv/bv* mice showed significantly higher numbers of GAD67-positive cells than control mice in the SC (Fig. 4B, *p* < 0.05) and AC (Fig. 4D, *p* < 0.05), but not in the ACC (Fig. 4A) or VC (Fig. 4C). The GAD67/PV double-positive cell count was also significantly higher in *bv/bv* mice than in control mice in the AC (Fig. 4D, *p* < 0.05), but not in the other areas (Fig. 4A–C). At 8 weeks of age, no significant differences were found between *bv/bv* and control mice in any cortices (Fig. 4). These findings suggest that, at an early stage of postnatal development, the number of GAD67-positive interneurons is not reduced in *bv/bv* mice; rather, it is mildly increased in the ACC and AC. Together with the lack of Srrm4 expression in the GABAergic interneurons, these findings indicate that the anxiety already present at 6 weeks of age is not associated with intrinsic abnormalities in GABAergic interneurons.

### 3.4 Impaired GABAergic synaptic activity in *bv/bv* mice

**Fig. 5:**
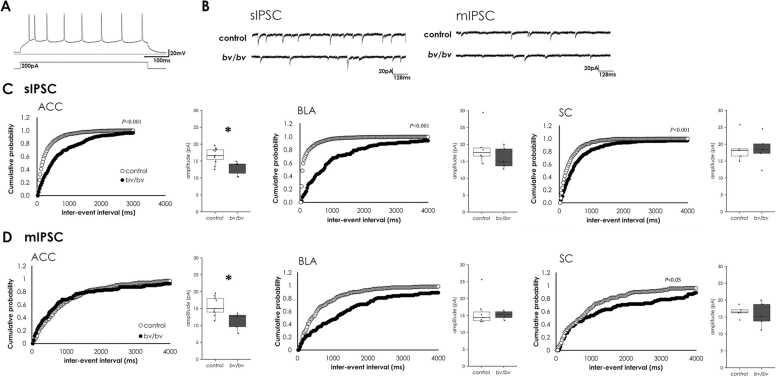
Whole-cell patch clamp recording of pyramidal neurons showed impaired GABAergic synaptic activity in *bv/bv* neurons. (A) Representative response of a *bv/bv* mouse pyramidal neuron to a square current pulse (200 pA) delivered via the recording electrode. Pyramidal neurons were identified and selected by spike-frequency adaptation based on morphology. (B) Representative traces of sIPSCs and mIPSCs in wild-type and *bv/bv* neurons from the ACC were shown. (C, D) Cumulative probability plots for inter-event intervals and box plots indicating the amplitude of sIPSCs (C) and mIPSCs (D) in the control (white) and *bv/bv* (black) mice from the ACC, BLA, and SC. ACC, auditory cortex; BLA, basolateral complex amygdala; SC, somatosensory cortex. n = 5–10 cells per genotype. Within each box, the horizontal line indicates the median value, and the boxes extend from the 25th to 75th percentile of the value distribution for each group. Vertical lines indicate the largest and smallest data points within 1.5 between the 25th and 75th percentile quartiles for each group. The dot plots indicate data for each mouse. * *p* < 0.05.

Next, we sought to examine whether the network between GABAergic interneurons and pyramidal neurons is functionally affected in *bv* mice. We examined GABAergic synaptic activity using whole-cell patch clamp recording of pyramidal neurons in acute brain slices. Pyramidal neurons were identified using spike-frequency adaptation (Fig. 5 A) in response to depolarizing pulses (Sah and Lopez De Armentia, 2003). We provoked action potential-induced GABAergic IPSCs (sIPSCs) in pyramidal neurons voltage-clamped at − 70 mV in the presence of CNQX, and MK-801 to block AMPA and NMDA receptors. We also analyzed miniature IPSCs (mIPSCs) in the presence of 0.5 µM TTX, which better indicates quantal release from individual synapses. At the end of recording, the IPSCs were completely blocked using 100 mM picrotoxin—a GABA_A_ receptor antagonist—to confirm that these discharges were mediated by GABA_A_ receptors.

The whole-cell patch clamp recording from the *bv/bv* mouse brain slices revealed significantly decreased sIPSC frequencies in ACC (control, n = 9 cells; *bv/bv*, n = 6 cells; *p* < 0.01), SC (control, n = 5 cells; *bv/bv*, n = 5 cells; *p* < 0.01), and basolateral complex amygdala (BLA; control, n = 6 cells; *bv/bv*, n = 5 cells; *p* < 0.01), suggesting that the frequency of GABA release was decreased in *bv/bv* mice. We also found that the amplitude of both sIPSCs and mIPSCs from ACC slices in *bv/bv* mice was significantly lower than in control mice (Fig. 5 C and D; sIPSC amplitude: control, n = 11 cells; *bv/bv*, n = 6 cells; *p* < 0.05; mIPSC amplitude: control, n = 9 cells; *bv/bv*, n = 5 cells; *p* < 0.05), indicating lower GABAergic synaptic conductance in the ACC of *bv/bv* mice. No significant change was observed in either the rise or decay time of sIPSCs and mIPSCs.

**Fig. 6:**
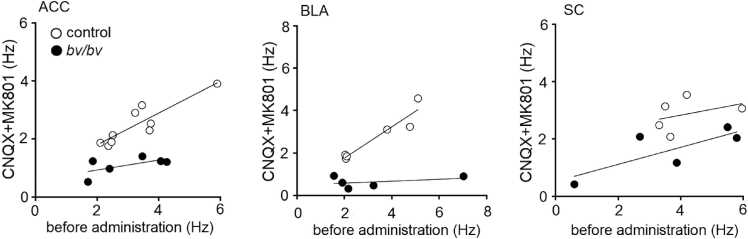
Scatter plots of baseline frequency against frequency in the presence of CNQX or MK-801. Scatter plots of baseline frequency versus sIPSC frequency for 5–10 neurons from the ACC, BLA, and SC. The lines indicate the approximate curve of frequencies with and without CNQX and MK801. ACC, auditory cortex; BLA, basolateral complex amygdala; SC, somatosensory cortex.

Scatter plots of baseline frequency against frequency in the presence of CNQX or MK-801 showed that, although there was no significant difference in baseline frequency between control and *bv/bv* mice, the frequency of IPSCs was significantly decreased in *bv/bv* mice (Fig. 6). These results suggest that, besides impaired IPSCs, excessive excitatory post-synaptic potential may also be observed in *bv/bv* neurons.

Furthermore, we examined GABA receptor functionality by examining seizure sensitivity to PTZ, which exerts a convulsive action through non-competitive antagonization of the GABA_A_ receptor complex (Ramanjaneyulu and Ticku, 1984). In all *bv/bv* and control mice, intraperitoneal administration of PTZ-induced seizures accompanied by hindlimb extension. The elapsed time to reach the first seizure in *bv/bv* mice (101 ± 12.0 s) was significantly shorter than in control mice (491 ± 104.7 s; *p* < 0.05). These results also suggest that there are abnormalities in GABAergic transmission in *bv/bv* mice.

**Fig. 7:**
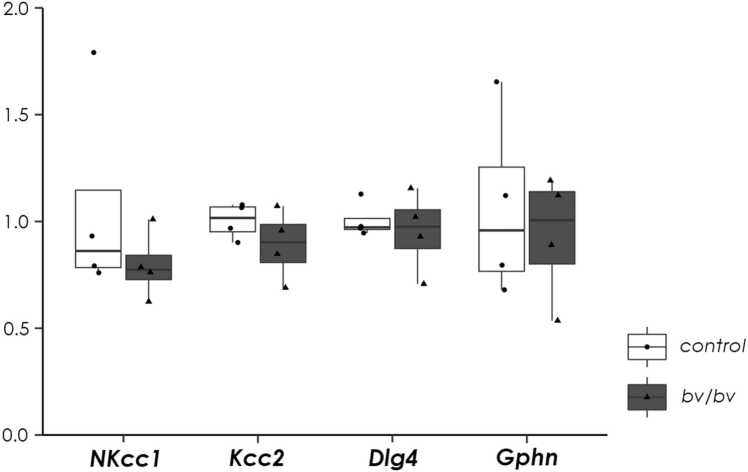
Evaluation of *Nkcc1*, *Kcc2*, *Dlg4*, and *Gphn* mRNA expression in the cortex using real-time PCR. All data were normalized to *GAPDH* expression levels (n = 4 per group). Within each box, the horizontal line indicates the median value, and the boxes extend from the 25th to 75th percentile of the value distribution for each group. Vertical lines indicate the largest and smallest data points within 1.5 between the 25th and 75th percentile quartiles for each group. The dot plots indicate data for each mouse.

We next examined the expression of two chloride co-transporter genes: *Nkcc1* and *Kcc2*, which are critical regulators of neuronal chloride concentration and are associated with the developmental transition of neuronal excitability from excitatory to inhibitory upon GABAergic input from interneurons. Kcc2 expression is negatively regulated by the RE1-silencing transcription factor (REST) (Yeo et al., 2009), whose alternative splicing is regulated by both Srrm4 and its paralog Srrm3 during neuronal maturation (Nakano et al., 2019). Quantitative RT-PCR showed no difference in *Nkcc1* or *Kcc2* mRNA levels between adult brains from *bv*/*bv* and wild-type mice, suggesting that the expression of these Cl^-^ transporters is balanced in the brain as a whole (Fig. 7). We also examined the expression of gephyrin gene (*Gphn*) and disks large homolog 4 gene (*Dlg4*), which are the synaptic scaffold proteins for glutamatergic and GABAergic neurons, respectively. We found no difference in either gene between *bv/bv* and wild-type mice, suggesting that no major synaptic structural abnormalities are present in *bv/bv* mice.

## 4 Discussion

### 4.1 *bv/bv* mice show anxiety-like behavior in the early postnatal period

Based on our previous observation that *bv*/*bv* mice show increased anxiety and a decreased number of GABAergic interneurons in specific areas of the cortex at 3–4 months of age (Matsuda et al., 2011), we hypothesized that *Srrm4* is expressed in the interneurons, and that the *bv* mutation in *Srrm4* results in either developmental failure or postnatal degeneration of interneurons.

Firstly, we determined when anxiety-like behavior becomes apparent in *bv*/*bv* mice. We found that anxiety is not evident at 3 weeks of age, but becomes apparent at 6 weeks of age, as we observed significant reduction in distance traveled in the open-arms of the elevated plus maze test, while we found no changes in locomotion, as measured by the open-field exploration test. We also confirmed that the anxiety persists through 3 months of age, as previously reported. Because Srrm4 regulates alternative splicing of neuronal transcripts, these findings suggest that proper splicing prevents mice from showing anxiety, and that *bv*/*bv* mice could be used as a model to study the effect of alternative splicing in anxiety disorders. One limitation in our behavior studies was that anxiety was evaluated by a single battery and no supplemental data were available due to technical reasons.

### 4.2 Srrm4 is barely expressed in GABAergic interneurons

Next, we determined the postnatal expression profile of *Srrm4* using in situ hybridization of *Srrm4* mRNA. Srrm4 continues to be broadly expressed in the neurons, but not in other types of cells, as evidenced by the lack of expression in the white matter, where oligodendrocytes, astrocytes, and microglia reside, but neurons are absent. Notably, in the diencephalon and mesencephalon, GABAergic interneurons did not collocate with neurons expressing *Srrm4* mRNA. In the telencephalon, it was technically difficult to clearly separate GABAergic interneurons and *Srrm4* signals using in situ hybridization, therefore, *Srrm4* expression in GABAergic interneurons at cellular level left undetermined a the cellular level in the telencephalon.

**Fig. 8:**
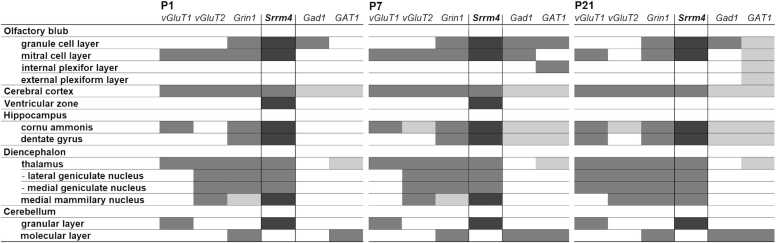
Developmental changes in regional expression of *Srrm4* mRNA and the marker genes for glutamatergic and GABAergic neurons. Expression for *Srrm4* was summarized based on the in situ hybridization data of this study. Expression profiles of *vGluT1*, *vGluT2*, *Grin*, *Gad1* and *GAT1* were obtained from several published studies (Miyazaki et al., 2003, Tamamaki et al., 2003, Fremeau et al., 2004, Lein et al., 2007, Masuho et al., 2008) and integrated into a comparable table.

Overall, our gross comparison of the *Srrm4* expression pattern with those of the glutamatergic neuron markers vesicular glutamate transporter genes (*vGluT1* and *vGluT2*) and glutamate ionotropic receptor NMDA type subunit 1 gene (*Grin1*), and of the GABAergic neuron markers, glutamate decarboxylase 1 gene (*Gad1*), and GABA transporter 1 gene (*GAT1*) indicated that the regions of glutamatergic neurons overlap with those containing *Srrm4*-expressing neurons, but that the regions of GABAergic interneurons do not (Fig. 8) (Miyazaki et al., 2003, Tamamaki et al., 2003, Fremeau et al., 2004, Lein et al., 2007, Masuho et al., 2008). These findings suggested that Srrm4 is barely expressed in GABAergic interneurons, but rather in glutamatergic neurons. Thus, our initial hypothesis that *Srrm4* is expressed in GABAergic interneurons was unsupported; instead, the *bv*-associated *Srrm4* mutation may cause anxiety by altering gene expression in glutamatergic neurons rather than in GABAergic interneurons.

### 4.3 Anxiety in *bv/bv* mice is not directly associated with reduction of interneurons

The inner ear cells in *bv*/*bv* mice appear to be lost by degeneration, which occurs in the perinatal to postnatal stages (Whitlon et al., 1996, Sobkowicz et al., 1999), suggesting that the effect of *Srrm4* mutation is degenerative and that it manifests soon after the completion of inner hair cell development. Therefore, we questioned whether the reduction in interneuron number found in the cortex of *bv/bv* mice at 3–4 months of age actually begins earlier, because *bv/bv* mice already exhibit anxiety at 6 weeks of age. However, contrary to this prediction, our investigation did not show any reduction in interneuron number at 3 or 8 weeks of age. Our results suggest that the reduced interneuron number observed 3 months after birth is likely a late onset event, and that it is not caused by the cell autonomous effect of the *Srrm4* mutation in interneurons. Thus, the exact reason for the late onset reduction of interneurons in *bv/bv* mice remains unknown. Nevertheless, we assume that the anxiety observed from an early age in *bv/bv* mice is not a direct consequence of a reduction in interneurons.

We observed an increase in GAD67-positive interneurons in the AC of *bv/bv* mice at both 3 weeks and 8 weeks, but not in other cortical regions. The reason for this lesion-specific change is unclear, but it may be that primary hearing loss affects the composition of neurons in the AC.

### 4.4 *Bv*/*bv* mice show functional abnormality of GABAergic interneurons

Although presynaptic GABAergic interneurons did not express Srrm4 and the number of these cells remained unchanged, anxiety phenotypes were already present at 6 weeks of age in *bv/bv* mice, suggesting that functional changes may have occurred in the interaction between presynaptic interneurons and postsynaptic neurons. When we examined the properties of sIPSCs, we found decreased amplitudes in ACC and reduced frequencies in both ACC and BLA of *bv/bv* mice, suggesting an abnormality in GABAergic neurotransmission. We next studied mIPSCs, which reflects the GABA response at single synaptic contacts or release sites (Gonzalez-Burgos et al., 2015), to test whether the changes in sIPSCs reflect changes in the quantal synaptic response to GABA. Similar to sIPSCs, the frequency of mIPSCs in BLA of *bv/bv* mice was significantly reduced in comparison with control mice (Fig. 5), which indicated a low synaptic release probability or decrease in the number of synaptic contacts. However, in ACC, the mIPSC frequency did not differ between *bv/bv* mice and control mice, which suggested that the decrease in sIPSCs frequency in ACC is the consequence of the decrease in release sites synchronously releasing GABA in an action potential-dependent manner. On the other hand, the amplitude of mIPSCs was lower in ACC of *bv/bv* mice than control mice. A change in mIPSC amplitude could result from a reduced amount of GABA packaged into single synaptic vesicle, or a dysfunction of postsynaptic receptors (Kilman et al., 2002). Since neither the rise time nor the decay time of sIPSCs and mIPSCs showed any changes, it was suggested that no gross qualitative changes occurred in the postsynaptic GABA receptors. These data suggest that the decrease in mIPSC amplitude may be due to a reduced amount of GABA in single synaptic vesicle. Meanwhile, our results also indirectly indicated an activity imbalance between the excitatory and inhibitory synapses (Fig. 6). All these data led us to hypothesize that *bv/bv* mice exhibit functional abnormalities in the interaction between presynaptic interneurons and postsynaptic neurons, which may underlie the anxiety in these mice.

We also observed that the elapsed time to PTZ-induced seizure was significantly shortened in *bv*/*bv* mice, which normally show no spontaneous seizure. PTZ reduces Cl^-^ conductance by binding to the benzodiazepine site of the GABA_A_ receptor, causing the neuronal excitability increase (Walsh et al., 1999, Rebrov et al., 2004). Although the increased sensitivity to PTZ-induced seizures does not specify the abnormality as either presynaptic or postsynaptic, it supports the notion that *bv*/*bv* mice show reduced GABAergic transmission, as suggested in the electrophysiological findings.

To further examine whether altered Cl^-^ concentrations could cause this susceptibility to PTZ-induced seizure, we investigated the expression of Nkcc1 and Kcc2, the latter being transcriptionally regulated by Srrm4 through its downstream transcription factor REST. Because Kcc2 is an outwardly-directed K^+^/Cl^-^ co-transporter, reducing its expression may increase the cellular Cl^-^ concentration, resulting in increased seizure susceptibility and potential anxiety, as observed in *bv*/*bv* mice. However, Srrm3 functions synergistically in the alternative splicing of Srrm4-target genes, including REST (Nakano et al., 2019). Therefore, Kcc2 expression may not be grossly altered in the brain of *bv*/*bv* mice, although it may be that Srrm3 expression is missing in a certain endogenous cell population; as such, Srrm4 may be indispensable for proper regulation of alternative gene splicing, including *Kcc2* splicing. In fact, single-cell RNAseq analysis has revealed such cell populations (Allen Cell Type Database, 2015). Further cell type-specific analysis in these cells may uncover the dysregulation of genes, including *Kcc2*, which is directly associated with seizure susceptibility and anxiety phenotypes in *bv*/*bv* mice.

These findings indicate that *Srrm4* alteration in *bv*/*bv* mice may result in GABA receptor dysfunction on the postsynaptic side, most likely affecting GABA_A_ receptors. Because the GABA_A_ receptor is associated with anxiety regulation (Löw et al., 2000, Raud et al., 2009), anxiety-like behaviors in *bv*/*bv* mice could be attributed to abnormities in postsynaptic GABA_A_ receptor functions. Alternatively, Srrm4 may regulate genes that contribute to the formation of inhibitory synapses. In such case, the lack of Smrr4 in pyramidal neurons may affect the inhibitory synapse formation.

### 4.5 Similarities and differences between *bv*/*bv* and *Srrm4* knockout mice

Our studies have demonstrated some similarities and differences between *bv/bv* and *Srrm4* knockout mice. Homozygous *Srrm4* knockout mice display head tilting and circling behavior, which are also observed in *bv/bv* mice. However, they also show reduced fertility (Quesnel-Vallières et al., 2015, Quesnel-Vallières et al., 2016), whereas *bv/bv* mice are fully viable, probably because the *bv* mutation preserve partial protein activity (Quesnel-Vallières et al., 2015, Nakano et al., 2012). These differences allow us to investigate *bv* mice in the postnatal and adult stages. Heterozygous *Srrm4* knockout mice show autistic-like features, such as abnormal social interaction (Quesnel-Vallières, et al., 2016). However, they show no anxiety-like behavior, whereas *bv*/*bv* mice do. Heterozygous *Srrm4* knockout mice also show electrophysiological abnormalities, with decreases in inhibitory synaptic transmission, similar to those seen in *bv*/*bv* mice. Therefore, different *Srrm4* genotypes appear to show variable phenotypic expressions, and only *bv*/*bv* mice could serve as a model for anxiety associated with *Srrm4*.

## 5 Conclusion

Our study showed that Srrm4 is not expressed in GABAergic interneurons, which appear to be intact in *bv*/*bv* mice when they show anxiety phenotype. Conversely, we found electrophysiological abnormalities in GABAergic transmission, probably because *bv/bv* mice have postsynaptic functional changes, as suggested in heterozygous *Srrm4* knockout mice (Quesnel-Vallières, et al., 2016). Given that Srrm4 is mainly expressed in pyramidal neurons, it may be involved in the postsynaptic regulation of GABAergic transmission, although we have not completely excluded presynaptic involvement, by controlling the alternative splicing of genes that are yet to be identified. Clarifying these genes may further delineate the molecular mechanisms by which *Srrm4* contributes to anxiety in *bv/bv* mice.

## Compliance with ethical standards

All experimental protocols were conducted in accordance with the guidelines for animal care and regulated by the animal committee of the National Institute of Neuroscience.

## Funding

This study was supported in part by the Intramural Research Grant for Neurological and Psychiatric Disorders of NCNP (grant numbers 23–7, 3–6, and 3–7), a Grant-in-Aid for Scientific Research (C) (grant number: C25461745) and Grant-in-Aid for Early-Career Scientists (grant number: 20K13967).

## CRediT authorship contribution statement

**Yuka Shirakawa**: Conceptualization, Methodology, Investigation, Formal analysis, Writing - Original Draft. **Heng Li**: Conceptualization, Methodology, Investigation, Formal analysis, Writing - Original Draft. **Yuki Inoue**: Conceptualization, Methodology, Investigation. **Hitomi Izumi**: Investigation. **Yoshimi Kaga**: Methodology. **Yu-ichi Goto**: Writing - Review & Editing. **Ken Inoue**: Conceptualization, Writing – Review & Editing, Supervision. **Masumi Inagaki**: Supervision, Funding acquisition.

## Declaration of Competing Interest

None.
